# Report-Level Impact of DL Assistance on Teleradiology Quality Support for Brain Metastases: Real-World Clinical Practice at a Single Tertiary Center

**DOI:** 10.3390/diagnostics16081211

**Published:** 2026-04-17

**Authors:** Jieun Roh, Hye Jin Baek, Seung Kug Baik, Bora Chung, Kwang Ho Choi, Hwaseong Ryu, Bong Kyeong Son

**Affiliations:** 1 Department of Radiology, Research Institute for Convergence of Biomedical Science and Technology, Pusan National University Yangsan Hospital, Pusan National University School of Medicine, 20 Geumo-ro, Mulgeum-eup, Yangsan-si 50612, Republic of Korea; 2 Department of Thoracic and Cardiovascular Surgery, Research Institute for Convergence of Biomedical Science and Technology, Pusan National University Yangsan Hospital, Pusan National University School of Medicine, 20 Geumo-ro, Mulgeum-eup, Yangsan-si 50612, Republic of Korea; 3 Department of Radiology, Pusan National University Yangsan Hospital, 20 Geumo-ro, Mulgeum-eup, Yangsan-si 50612, Republic of Korea

**Keywords:** brain metastases, teleradiology, deep learning, magnetic resonance imaging, quality control

## Abstract

**Objective:** Existing deep learning (DL) studies on brain metastasis have largely focused on algorithm or reader performance in controlled settings, whereas its role in routine teleradiology quality support remains unestablished. We evaluated the report-level impact of DL assistance on brain metastasis interpretation in a real-world teleradiology workflow using dual-sequence MRI. **Materials and Methods**: In this retrospective study, 600 patients who underwent contrast-enhanced dual-sequence brain MRI during two consecutive 3-month periods before (pre-DL, n = 286) and after (post-DL, n = 314) DL integration into teleradiology workflow were analyzed. Ten board-certified teleradiologists interpreted all the cases with or without DL-generated overlays. Report-level diagnostic metrics were assessed against a consensus reference standard established by faculty neuroradiologists. Subsequently, exploratory case-level stratified sensitivity analyses were performed for metastasis-positive examinations based on lesion multiplicity and the largest lesion size. Teleradiologists’ perceptions were assessed using a post-interpretation survey. **Results:** Compared with the pre-DL group, the post-DL group showed higher sensitivity (77.7% vs. 90.8%, *p* < 0.001), specificity (82.3% vs. 90.8%, *p* = 0.002), accuracy (80.8% vs. 90.8%, *p* < 0.001), positive predictive value (68.2% vs. 85.7%, *p* < 0.001), and negative predictive value (88.3% vs. 94.2%, *p* = 0.011). False-positive and false-negative rates were lower after DL implementation (11.9% vs. 5.7%, *p* = 0.009; 7.3% vs. 3.5%, *p* = 0.045). Sensitivity gains were most pronounced for cases with single metastasis (74.6% vs. 91.2%, *p* = 0.007) and with the largest lesion ≤ 5 mm (74.3% vs. 92.0%, *p* = 0.004), whereas sensitivity was similar for multiple metastases and for cases with a largest lesion > 5 mm. Survey responses suggested favorable usability and diagnostic support. **Conclusions**: In this real-world teleradiology workflow, DL implementation was associated with higher report-level diagnostic metrics and fewer false interpretations. DL assistance may help support quality control for brain metastasis interpretation, particularly in more subtle and diagnostically challenging cases, although radiologist judgment remains essential for subtle or borderline lesions.

## 1. Introduction

Brain metastases are common in patients with systemic cancer and critically influence their prognosis and therapeutic planning [1]. Accurate detection, quantification, and longitudinal monitoring of these lesions are essential to determine individualized treatment strategies [2]. Contrast-enhanced magnetic resonance imaging (MRI) is the gold standard for detecting brain metastases. However, dual-sequence protocols combining white (e.g., MPRAGE) and black (e.g., SPACE/CUBE) blood imaging are increasingly being integrated with deep learning (DL) algorithms for automated lesion detection and segmentation because they enhance lesion conspicuity [3,4,5,6].

Recent studies have reported promising results for DL-based brain metastasis detection, especially in controlled research settings. A data-centric DL model trained and validated by Topff et al. across multiple institutions demonstrated high sensitivity and generalizability for detecting brain metastases on contrast-enhanced MR images [7]. Similarly, Luo et al. conducted a multi-center randomized crossover study and reported the benefits of artificial intelligence (AI) assistance in improving the accuracy and efficiency of brain metastasis segmentation by radiologists in controlled academic settings [8]. Other studies have reported the sensitivity and positive predictive value of DL tools trained on dual-sequence MR images for detecting brain metastases across single- and multi-center cohorts [9,10]. Collectively, these recent studies present critical benchmark evidence for the technical feasibility and diagnostic potential of DL-based assistance for brain metastasis detection. However, they have primarily focused on algorithmic performance or reader-assistance effects in controlled in-house or research settings rather than on the report-level impact of DL support in routine outsourced teleradiology practice.

Despite these technological advances, interpreting brain metastases remains challenging due to varying lesion characteristics that contribute to radiologists’ attention fatigue [11]. This challenge is amplified by faculty shortages in academic and non-academic settings, creating a global workforce crisis [12]. To overcome such faculty shortage, many institutions rely on teleradiology services, which can improve access and turnaround time, particularly in high-volume tertiary care settings [13,14]. In addition, routine practice is challenged by persistent quality gaps, including missed diagnoses, false positives, variable subspecialty expertise, and reporting inconsistencies that may affect patient care and medicolegal accountability [15,16,17,18,19,20]. In many real-world practice environments, partial reliance on such outsourced services may be operationally difficult to avoid, especially when sustained in-house subspecialty coverage cannot be maintained. Nevertheless, evidence regarding whether DL can provide practical report-level quality support in such settings, where interpretations are often generated under routine clinical conditions rather than within structured research frameworks, remains limited. Furthermore, a recent multi-society study has also emphasized that the clinical value of AI in radiology depends not only on technical performance but also on how such tools are implemented, monitored, and integrated into real-world workflows [21]. Therefore, beyond algorithmic accuracy alone, the clinically relevant question is whether DL can function as a practical quality-support mechanism within routine teleradiology interpretation in everyday practice.

Our institution uses a dual-sequence MRI protocol (MPRAGE and black-blood imaging) for brain metastasis evaluation across both in-house neuroradiology and vendor-based teleradiology practice. Although report quality remains clinically consequential, systematic lesion-level audit is limited because external reports are generated in routine free-text form and interpreted by radiologists with heterogeneous backgrounds. Therefore, we evaluated whether implementation of a DL-assisted detection tool was associated with report-level changes in teleradiologists’ interpretations within a real-world outsourced teleradiology workflow, and whether DL assistance may serve as a practical adjunct for quality support in this setting.

## 2. Materials and Methods

### 2.1. Study Design and Patient Selection

**Figure 1:**
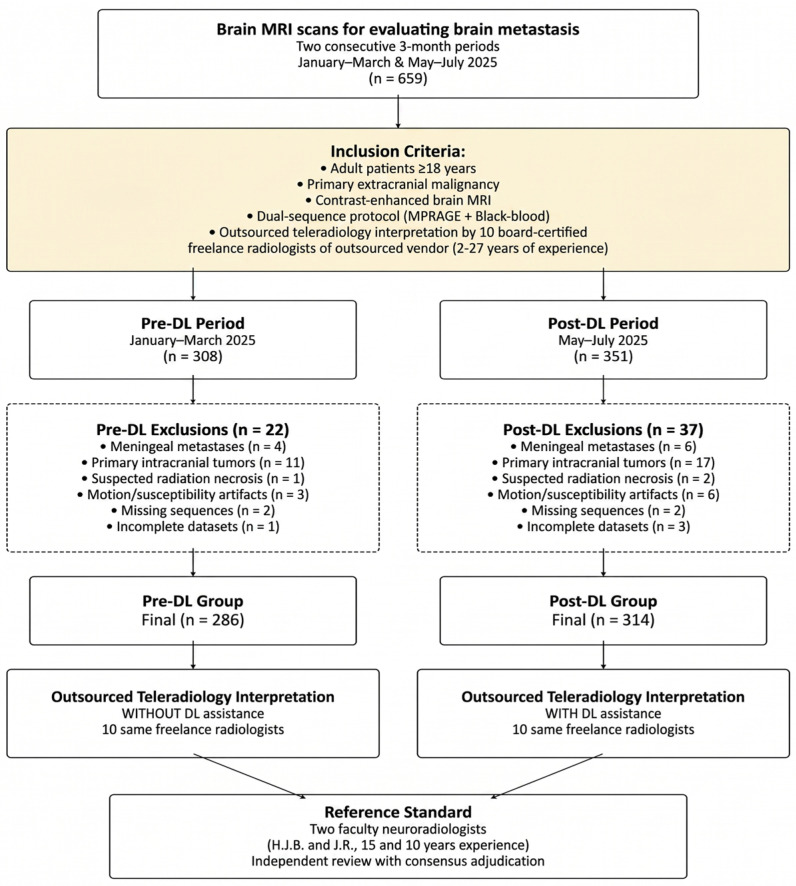
Flowchart of the patient inclusion process.

This retrospective study was conducted at a tertiary academic referral center. The study was reviewed by the Institutional Review Board of Pusan National University Yangsan Hospital, which exempted the study (IRB No. 55-2025-111; Date of exemption determination: 17 September 2025) from the need for any approval or informed consent. Patients were divided into pre-DL (January–March 2025) and post-DL (May–July 2025) groups based on whether brain MRIs were acquired before or after incorporation of the DL-assisted detection tool into our outsourced teleradiology workflow. The month of April 2025 was treated as a predefined transition period for workflow integration and radiologist adaptation to the DL-assisted system, and this period was excluded from cohort selection. The patients (aged ≥18 years) with known primary extracranial malignancies who underwent contrast-enhanced brain MRI for brain metastasis evaluation were included in this study. The exclusion criteria comprised presence of meningeal metastases, primary intracranial tumors, suspected radiation necrosis, significant motion or susceptibility artifacts; absence of white blood or black blood contrast-enhanced T1-weighted sequences; and incomplete imaging datasets. The patient selection process is illustrated in Figure 1.

### 2.2. MRI Acquisition Protocol

All examinations were performed using 3T MR systems (SIGNA™ Premier; GE Healthcare, Waukesha, WI, USA; MAGNETOM Skyra; Siemens Healthineers, Erlangen, Germany; MAGNETOM Vida; Siemens Healthineers, Erlangen, Germany) with 32-, 48-, or 64-channel head coils. The institutional protocol comprised contrast-enhanced dual-sequence acquisition with white-blood (MPRAGE) and black-blood (SPACE or CUBE) T1-weighted imaging according to consensus recommendations [22] (Table 1). The sequences were acquired 5 min after intravenous administration of a gadolinium-based contrast agent (0.1 mL/kg of gadobutrol [Gadovist, Bayer Schering Pharma] or gadoterate meglumine [Dotarem, Guerbet]) and were reconstructed in the axial, sagittal, and coronal planes.

### 2.3. DL-Based Detection System

The DL-based detection system utilized a commercially developed 3D nnU-Net-based DL architecture designed to detect brain metastases on contrast-enhanced dual-sequence MRI (3D gradient-echo and 3D turbo spin-echo with black-blood technique). Various multicenter studies have reported a high performance and reproducibility of this approach, with both sensitivity and positive predictive values exceeding 90% [5,6]. The network was originally trained on a separate multicenter dataset from different institutions, comprising 200 patients with 503 brain metastases, which was not included in the study’s clinical cohort.

The training and validation framework followed previously described configurations [6,23], implemented with the open-source nnU-Net framework (https://github.com/MIC-DKFZ/nnUNet (accessed on 1 January 2026)) in PyTorch 1.1 (Python 3.7) and HD-BET for skull stripping (https://github.com/MIC-DKFZ/HD-BET (accessed on 1 January 2026)). Compared with conventional U-Net, nnU-Net automatically configures preprocessing, architecture, and hyperparameters according to dataset fingerprint (e.g., class ratio, image size, voxel spacing), thereby optimizing performance for clinical use [6]. A full-resolution 3D architecture was adopted instead of 2D or cascade models because brain-metastatic lesions are typically small, simple-shaped, and often multiple. For each patient, co-registered TSE and GRE image pairs were fed into the network. Training employed Dice + cross-entropy loss, Adam optimizer (initial learning rate 0.0003 with weight decay), patch size 128 × 128 × 112, batch size 2, and data augmentation (rotation, gamma, scaling, elastic deformation, and mirror transforms). Model convergence occurred after approximately 507 epochs over 2.5 days on an NVIDIA TITAN RTX 24 GB GPU with CUDA 10.0 [6].

The final trained DL model used in this study corresponded to a commercially developed model currently under FDA review, integrated into the institutional PACS environment to provide automated lesion detection with colored overlay markers for teleradiology reading. Because the software is proprietary and under regulatory evaluation, its trained weights and source code cannot be publicly released. Nevertheless, the training configuration and algorithmic framework are reproducible from the cited publications and open-source components, and the corresponding author can provide additional technical details upon reasonable request. Radiologists reviewed and verified all overlays in real time, maintaining full interpretive responsibility. Black-blood imaging effectively suppressed vascular signals, reducing false-positive detections and improving interpretability [6,23].

### 2.4. Image Interpretation and Teleradiology Workflow

**Figure 2:**
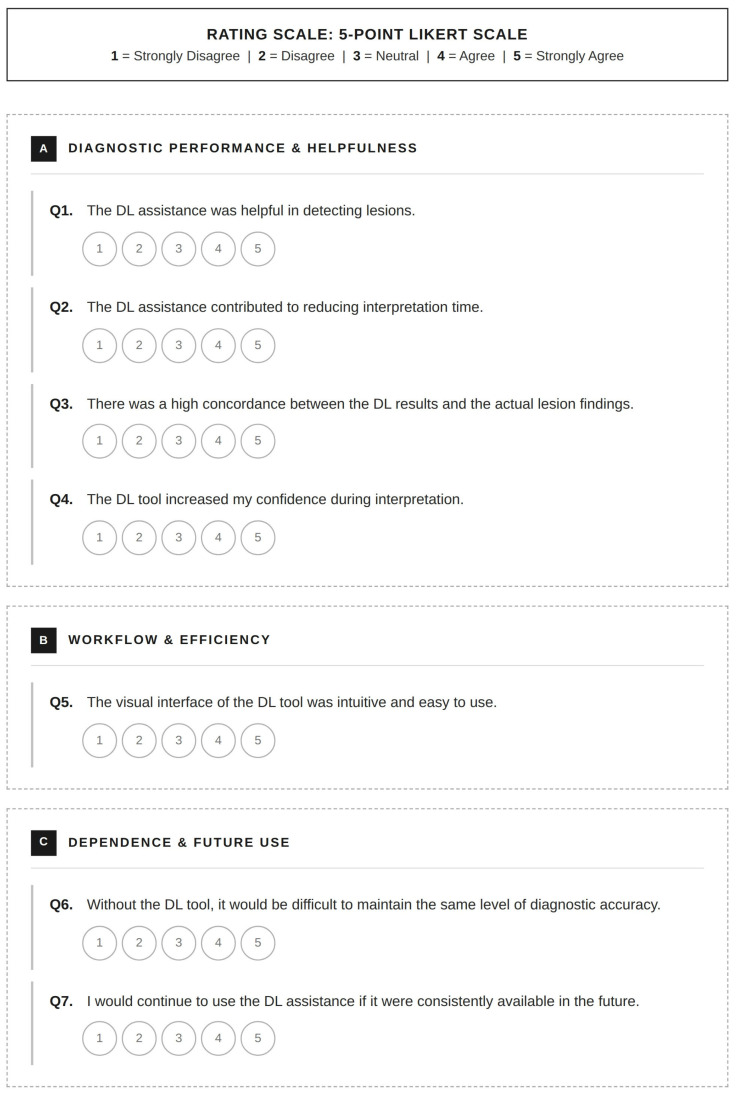
Survey questionnaire for radiologists’ perception of DL-assisted interpretation. Responses were recorded on a 5-point Likert scale ranging from 1 (Strongly Disagree) to 5 (Strongly Agree). Items were grouped into three domains: (**A**) diagnostic performance and helpfulness, (**B**) workflow and efficiency, and (**C**) dependence and anticipated future use. DL = deep learning.

The images were interpreted by 10 board-certified teleradiologists working with a teleradiology vendor. Their post-certification experience ranged from 2 to 27 years (mean, 8.5 years); among these, 4 (40%), 4 (40%), 1 (10%), and 1 (10%) teleradiologists had ≤5, 6–10, 11–20, and >20 years of experience, respectively. Two radiologists (20%) were fellowship-trained in neuroradiology. Each examination was interpreted by a single assigned teleradiologist according to routine clinical workflow, generating one final clinical report; no multi-reader re-interpretation was performed for the purpose of this study. Reader assignment followed the vendor’s routine scheduling practice and was not controlled by the investigators, as the purpose was to evaluate DL implementation under real-world outsourced teleradiology conditions. All radiologists had access to relevant clinical information and prior imaging studies, when available. The radiologists interpreted the dual-sequence MR images of the pre-DL group without AI assistance. In the post-DL period, DL-generated detection overlays were automatically displayed within the PACS viewer for every examination, and radiologists could incorporate or disregard the overlays at their discretion for the final interpretation. The primary endpoints were report-level quality outcomes derived from the teleradiology reports: (1) the reported presence or absence of brain metastases and (2) the reported lesion multiplicity. The presence of brain metastases was recorded only when explicitly stated in the report. Multiplicity was recorded as positive if it was reported based on descriptions such as “multiple metastases” or “numerous enhancing foci.” Lesions not meeting these criteria were considered single. Information on lesion size, location, and confidence level was inconsistently documented in the free-text reports; hence, it was excluded from the analysis. Because the external reports were not structured for lesion-by-lesion annotation, reliable retrospective lesion-level matching between the teleradiology reports and the reference standard was not feasible. Radiologists’ perceptions of DL assistance were assessed using a 7-item survey questionnaire. Their responses were recorded on a 5-point Likert scale (1 = strongly disagree to 5 = strongly agree). The questionnaire assessed parameters such as usability, diagnostic support, trust in DL results, and future acceptance. The detailed structure of the survey questionnaire is illustrated in Figure 2.

### 2.5. Reference Standard

Two faculty neuroradiologists with 15 and 10 years of neuroradiology experience, blinded to the teleradiology interpretations and DL outputs, independently classified the lesions based on established criteria for brain metastases. Pre-consensus inter-reader agreement between the two reference reviewers was assessed. Initial discrepancies or equivocal findings were documented and systematically resolved during consensus sessions. Following IRB approval, follow-up MRI obtained 3–6 months later in routine clinical care was retrospectively reviewed for selected ambiguous lesions, when available, to support final classification. No lesions were retained as indeterminate in the final reference standard. A senior neuroradiologist with 35 years of neuroradiology experience served as adjudicator when consensus could not be reached. Because histopathological confirmation was predominantly unavailable, expert consensus interpretation supplemented by available short-term follow-up imaging in selected cases served as the reference standard for evaluating diagnostic performance. The metastatic lesions were categorized based on their maximum axial diameters on the post-contrast images: ≤5 mm or >5 mm. The lesion size and multiplicity were determined by the reference reviewers because they were inconsistent in the external reports.

### 2.6. Statistical Analysis

Continuous variables are presented as mean ± standard deviation and were compared using independent *t*-tests. Categorical variables are summarized as counts and percentages and were compared using chi-squared or Fisher’s exact test. Effect sizes were calculated using Cohen’s d for continuous and Cramér’s V for categorical variables. Sensitivity, specificity, positive predictive value, negative predictive value, and accuracy were calculated with 95% confidence intervals and compared using two-tailed Z-tests for independent proportions. Inter-reader agreement between the two reference neuroradiologists prior to consensus was assessed using Cohen’s kappa statistic. Agreement between the DL-assisted diagnosis and reference standard was evaluated using Cohen’s kappa statistic and McNemar’s test. All primary analyses were performed at the examination/report level. Because the study was designed as a pragmatic workflow evaluation rather than a multi-reader multi-case experiment, reader-level clustering was not explicitly modeled. Exploratory case-level stratified sensitivity analyses were additionally performed among metastasis-positive examinations according to reference-standard lesion multiplicity and the largest lesion size, using Fisher’s exact test because of small subgroup counts. Statistical analyses were performed using Python (version 3.10.12; SciPy and StatsModels packages) and MedCalc (version 23.3.1). A two-sided *p* < 0.05 was considered statistically significant.

## 3. Results

### 3.1. Study Population: Pre-DL vs. Post-DL Groups

Of the 600 patients included in the study, 286 and 314 were allocated to the pre-DL and post-DL groups, respectively. The patient selection process is illustrated in Figure 1. The patients in the post-DL group were significantly older than those in the pre-DL group (67.2 ± 10.0 vs. 60.5 ± 15.0 years, *p* < 0.001). Regarding patients’ characteristics, sex and primary cancer type distributions in the groups were similar, and lung cancer was the most common (83.6% vs. 82.8%) cancer type. Regarding the prevalence of brain metastases, the two groups were comparable (pre-DL: 32.9% vs. post-DL: 37.9%, *p* = 0.230). Single metastases were more frequent than multiple metastases in both groups (pre-DL: 71.3% vs. post-DL: 76.5%, *p* = 0.484). Small lesions (≤5 mm) accounted for approximately three-quarters of the cases in both groups (pre-DL: 74.5% vs. post-DL: 73.9%, *p* = 0.920). The clinical characteristics are provided in Table 2.

### 3.2. Report-Level Diagnostic Metrics and Intergroup Comparison of Teleradiology Interpretations: Pre-DL vs. Post-DL Groups

Diagnostic metrics were lower in the pre-DL group compared with those in the post-DL groups (Table 3); sensitivity increased from 77.7% to 90.8% (*p* < 0.001), specificity increased from 82.3% to 90.8% (*p* = 0.002), and accuracy increased from 80.8% to 90.8% (*p* < 0.001). With DL implementation, the positive predictive value improved from 68.2% to 85.7% (*p* < 0.001), and the negative predictive value increased from 88.3% to 94.2% (*p* = 0.011), corresponding to absolute gains of 13.1 percentage points in sensitivity, 8.5 in specificity, 17.5 in positive predictive value, 5.9 in negative predictive value, and 10.0 in accuracy.

**Figure 3:**
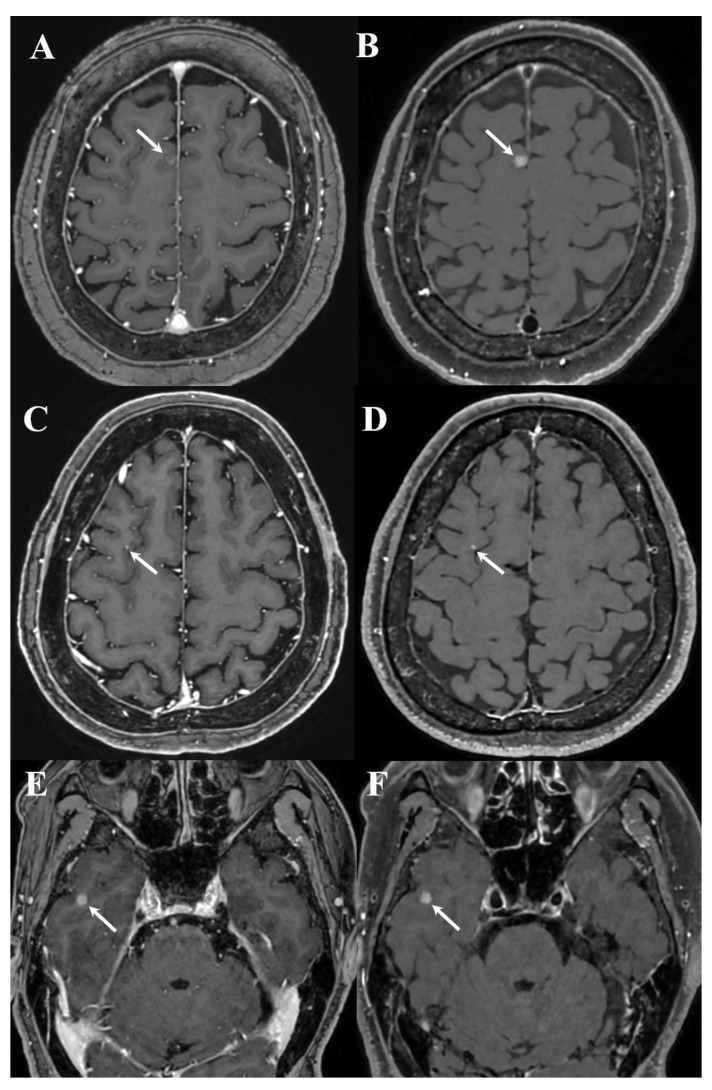
Examples of false negative cases on dual-enhanced T1-weighted images (T1WI) in pre-DL group ((**left**) column: white blood T1WI, (**right**) column: black blood T1WI). A teleradiologist missed brain metastases (arrows) in the right high frontal lobe ((**A**,**B**): superior frontal gyrus; (**C**,**D**): middle frontal gyrus), and the inferior portion of the right temporal lobe (**E**,**F**).

**Figure 4:**
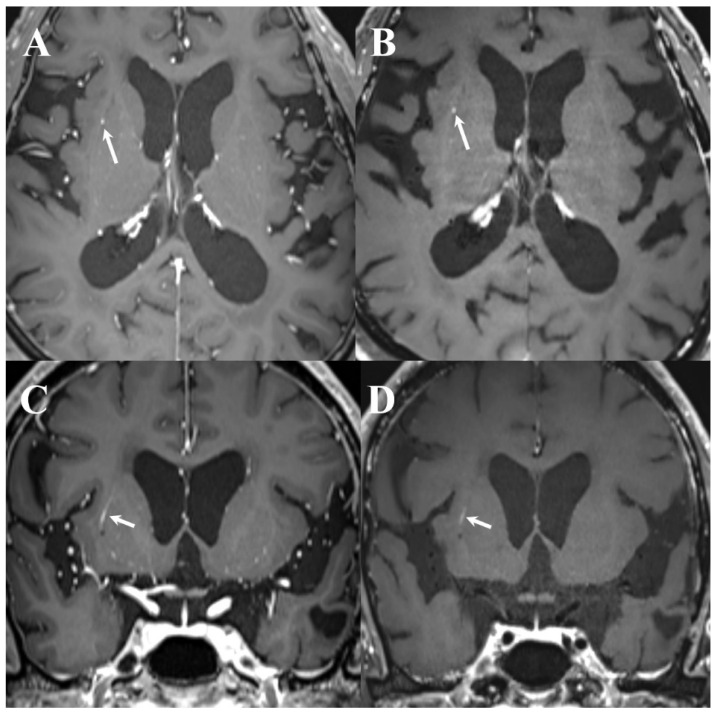
Example of a false positive case on dual-enhanced T1-weighted images (T1WI) in pre-DL group ((**left**) column: white blood T1WI, (**right**) column: black blood T1WI). A teleradiologist detected a small enhancing nodular lesion in the right basal ganglia (arrows in (**A**,**B**)); however, the lesion represented normal vascular enhancement on coronal T1WI (arrows in (**C**,**D**)).

**Figure 5:**
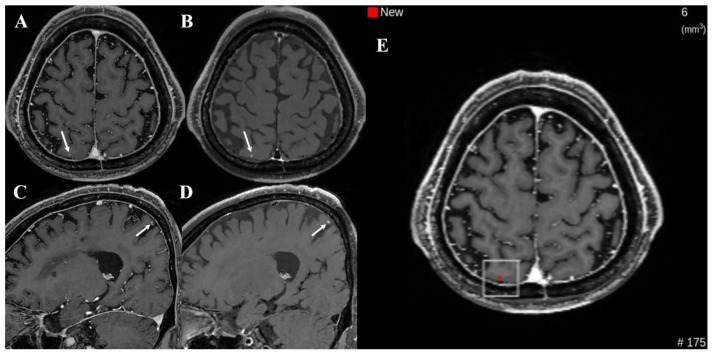
Example of a false negative case on dual-enhanced T1-weighted images (T1WI) in the post-DL group ((**left**) column: white blood T1WI, (**right**) column: black blood T1WI). A teleradiologist missed brain metastases (arrows in (**A**–**D**)) in the right parietal lobe even though DL detected the small brain metastasis (white box in (**E**)).

**Figure 6:**
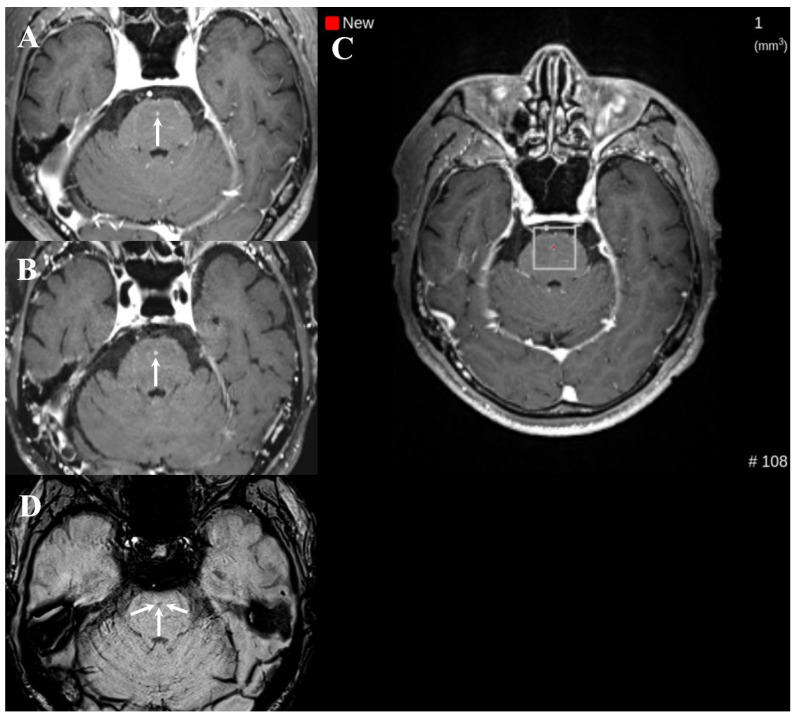
Example of a false positive case on dual-enhanced T1-weighted images (T1WI) in the post-DL group. Both the teleradiologist and DL considered a tiny enhancing nodular lesion as brain metastasis (arrows in dual-enhanced T1WI (**A**,**B**); and white box in (**C**)). However, the lesion shows focal low signal intensity on susceptibility-weighted image (arrows in (**D**)), suggesting a low-flow vascular malformation such as capillary telangiectasia.

**Figure 7:**
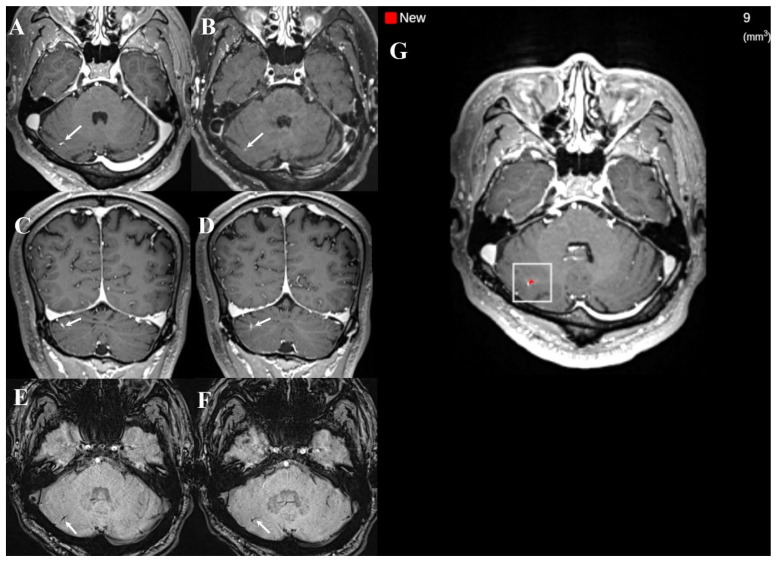
Example of a false positive case on DL detection only in the post-DL group. A small developmental venous anomaly (DVA) is noted in the right cerebellar hemisphere on dual-enhanced T1-weighted images (T1WI) (arrows in (**A**–**D**); (**A**,**C**): white blood T1WI; (**B**,**D**): black blood T1WI), showing linear low signal intensity on susceptibility-weighted imaging (arrows in (**E**,**F**)). The teleradiologist correctly identified the lesion as DVA, whereas DL incorrectly classified the lesion as brain metastasis (white box in (**G**)).

Further, error rates were lower in the post-DL group. With DL implementation, the false-positive rate decreased from 11.9% (34/286) to 5.7% (18/314) (*p* = 0.009), and the false-negative rate decreased from 7.3% (21/286) to 3.5% (11/314) (*p* = 0.045). These changes corresponded to absolute reductions of 6.2 percentage points in the false-positive rate and 3.8 percentage points in the false-negative rate. Representative cases for both periods are shown in Figure 3, Figure 4, Figure 5, Figure 6 and Figure 7.

Among metastasis-positive examinations, exploratory case-level stratified analyses showed that sensitivity improvement was more pronounced for single metastases (74.6% [50/67] vs. 91.2% [83/91], absolute difference + 16.6 percentage points, *p* = 0.007) and for cases with the largest lesion ≤ 5 mm (74.3% [52/70] vs. 92.0% [81/88], absolute difference + 17.8 percentage points, *p* = 0.004). In contrast, sensitivity remained similar for cases with multiple metastases (85.2% [23/27] vs. 89.3% [25/28], *p* = 0.705) and for cases with the largest lesion > 5 mm (87.5% [21/24] vs. 87.1% [27/31], *p* = 1.000).

### 3.3. Agreement Analyses: Reference Reviewers, Output by DL vs. Teleradiologists vs. Reference Standard

Before consensus, the two reference neuroradiologists showed almost perfect agreement for lesion classification (overall: Cohen’s κ = 0.963, 95% CI 0.941–0.986, *p* < 0.001; pre-DL: κ = 0.968, 95% CI 0.937–0.999, *p* < 0.001; post-DL: κ = 0.959, 95% CI 0.927–0.992, *p* < 0.001). Agreement was evaluated using Cohen’s kappa and McNemar tests for three comparisons: (1) DL output vs. teleradiologists’ interpretations, (2) DL output vs. the reference standard, and (3) teleradiologists’ interpretations vs. the reference standard. All kappa values were statistically significant (*p* < 0.001). DL output and teleradiologists’ interpretations showed almost perfect agreement (κ = 0.882), with no significant discordance (McNemar test, *p* = 0.099). DL output showed substantial agreement with the reference standard (κ = 0.769), while yielding more positive findings (McNemar test, *p* = 0.018). Further, teleradiologists’ interpretations showed substantial agreement with the reference standard (κ = 0.806), with no significant discordance (McNemar test, *p* = 0.265).

### 3.4. Survey of Outsourced Teleradiologists on DL Assistance (Exploratory)

Eight (80%) of the 10 participating radiologists completed the post-interpretation survey. Overall, mean item scores were above 4.0 on a 5-point Likert scale for perceived usability and diagnostic support (Table 4), including lesion detection assistance (mean, 4.88) and interface usability (mean, 5.00). Items related to long-term reliance showed greater variability and lower mean scores (mean, 3.75). Given the small sample size, the survey findings are descriptive and exploratory.

## 4. Discussions

We evaluated report-level interpretation outcomes before and after DL implementation in a real-world teleradiology workflow for brain metastasis evaluation using dual-sequence MRI. At the group level, report-level sensitivity, specificity, positive predictive value, negative predictive value, and accuracy were higher, and both false-positive and false-negative interpretations were less frequent in the post-DL group compared with those in the pre-DL group. Rather than serving as a direct efficacy test of the algorithm itself, these data provide a pragmatic view of how DL assistance may function within a teleradiology interpretation environment characterized by heterogeneous reader expertise and routine free-text reporting.

Notably, the false positives decreased from 11.9% in the pre-DL group to 5.7% in the post-DL group. One plausible explanation is that the dual-sequence input (MPRAGE and black-blood imaging), which reportedly improves the discrimination between metastases and vascular mimics, may have contributed to fewer vascular-related false calls in routine interpretation [3,4,5,6]. Similarly, cross-sequence information may have helped reduce single-sequence pitfalls [3,4,5,6]. Despite these differences, false negatives persisted for small (≤5 mm) and/or solitary lesions with faint enhancement (Figure 3, Figure 4 and Figure 5). Exploratory case-level stratified analyses supported these interpretations; notably, the sensitivity gains were most pronounced in cases with single metastases and in those with the largest lesion ≤ 5 mm, whereas sensitivity for examinations with multiple metastases or the largest lesion > 5 mm remained consistently high across both groups, showing no statistically significant change. These findings suggest that the clinical impact of DL assistance may be greatest in more subtle and diagnostically challenging cases, where perceptual misses are more likely. Because the analysis was based on report-level outcomes rather than on direct assessment of reader behavior, the reasons for missed subtle lesions cannot be determined; however, they may relate to a combination of technical (e.g., lesion conspicuity) and workflow-related reader (e.g., time pressure or variable reliance on DL cues) factors.

The DL system occasionally flagged benign low-flow vascular lesions (e.g., developmental venous anomaly and capillary telangiectasia) as metastases, as reflected in the McNemar test results (Figure 6 and Figure 7). Although dual-sequence information is intended to distinguish between metastases and vascular mimics, certain vascular-related findings remained challenging, underscoring the need for radiologists’ oversight. Further performance optimization may require algorithmic refinement (e.g., additional training data enriched for vascular mimics) and workflow-oriented safeguards. In outsourced radiology, inconsistent use of multiplanar reconstructions or ancillary sequences—when not routinely incorporated into the reading workflow—may further limit reliable characterization of such borderline findings.

In outsourced radiology, brain metastasis interpretation is inherently vulnerable to inter-reader variability because examinations are not consistently interpreted by the same radiologist and reader backgrounds are heterogeneous. In our study, only 20% of participating teleradiologists were fellowship-trained in neuroradiology, and post-certification experience ranged 2–27 years, indicating substantial variation in expertise. Such variability in training and experience could have influenced the baseline report-level performance observed in the pre-DL group [11]. Because reader allocation was not standardized and reader-level clustering was not explicitly modeled, part of the observed report-level difference may reflect between-reader heterogeneity in addition to workflow-level DL implementation. Nevertheless, after implementation of DL overlays, diagnostic metrics were higher and error rates were lower at the group level, supporting DL use as a quality-support adjunct for heterogeneous readers. At the same time, persistent false negatives and discordant cases underscore the expertise of radiologists, particularly for subtle or context-dependent findings.

Recent studies have reported promising role of DL systems for brain metastasis detection in controlled research settings. Topff et al. reported a DL model with high sensitivity and generalizability across multiple institutions [7], and Luo et al. reported improved reader performance and efficiency with AI assistance in an in-house, multi-reader setting [8]. Unlike such structured benchmark settings, our study relied on routine free-text teleradiology reports, thereby precluding lesion-level performance estimates. Instead, our study complements existing literature by providing report-level observations from routine outsourced radiology after clinical implementation of DL overlays. Accordingly, our findings demonstrate practice-based evidence regarding workflow-level quality support and real-world implementation of AI in teleradiology, rather than as direct causal estimates of DL efficacy [14,24].

In this real-world teleradiology workflow, higher report-level diagnostic metrics and lower error rates in the post-DL group suggest that DL overlays may aid in lesion detection and serve as a practical quality-support mechanism in teleradiology interpretation. This is particularly relevant in vendor-based workflows where heterogeneous expertise and non-standardized free-text reporting can limit systematic quality surveillance. Although the downstream clinical impact cannot be determined from our design, our experience suggests that DL assistance may help narrow report level variability and be effective for quality control in contemporary teleradiology practice. Future prospective and lesion-level studies should clarify how such tools can be integrated with reader training, workflow alignment, and structured auditing strategies.

This study has several limitations. Notably, the patients in the post-DL group were older than those in the pre-DL group, reflecting a between-group difference in baseline characteristics. However, the prevalence of brain metastases and reference-standard lesion features, including lesion multiplicity and lesion size, were comparable between the two groups. Although metastasis prevalence and reference-standard lesion features were comparable, residual confounding cannot be excluded in this non-paired design. Further, as a retrospective comparison of two independent, non-paired cohorts without randomization, residual confounding related to patient characteristics, case mix, temporal effects, and reader-level factors cannot be excluded. Because examinations were interpreted by multiple teleradiologists under routine vendor scheduling rather than by investigator-controlled allocation, and reader-level clustering was not explicitly modeled, between-reader heterogeneity might have contributed to the report-level variance. Furthermore, although a predefined one-month transition period was excluded from the study period to reduce immediate workflow adaptation effects, stable reader adaptation to DL assistance could not be directly verified. Accordingly, the findings should be interpreted as practice-based, report-level observations associated with DL implementation rather than as causal estimates of DL efficacy. Moreover, the number of participating teleradiologists was limited, and their heterogeneous training backgrounds may restrict generalizability to other teleradiology settings. In addition, the reference standard was based primarily on expert consensus without routine histopathologic confirmation or standardized follow-up for the entire cohort. Although the two reference neuroradiologists showed almost perfect pre-consensus agreement, and available 3–6-month follow-up MRI was retrospectively reviewed for selected ambiguous lesions, such follow-up was not available for all cases. Therefore, some degree of misclassification has remained, particularly for very small or equivocal enhancing lesions. Nevertheless, this approach remains practical and commonly used in imaging AI studies [3,5,6,23]. In addition, because ancillary sequence utilization and reading behaviors were not directly assessed, workflow-related contributors to false-positive or false-negative interpretations remain speculative. Finally, lesion-level analysis was not feasible because the outsourced teleradiology reports were written in routine free-text format and did not consistently document lesion number, size, location, or confidence for individual lesions. This limitation restricts direct assessment of lesion-level detection performance and may have introduced information bias. We considered the feasibility of a subset lesion-level analysis; however, because lesion-level information was available only inconsistently, restricting the analysis to a partially documented subset would have introduced additional selection bias. Future prospective studies using structured reporting or dedicated lesion-level auditing are needed to evaluate lesion-level performance.

## 5. Conclusions

In this single-center real-world teleradiology setting using dual-sequence MRI, report-level diagnostic metrics were higher and error rates were lower after DL implementation. These findings support the potential role of DL assistance as a practical adjunct for quality support in teleradiology brain metastasis interpretation across readers with heterogeneous expertise, particularly in more subtle and diagnostically challenging cases, including those with small and/or solitary metastatic lesions. Nonetheless, radiologist judgment remains essential for subtle or borderline findings. Further studies—including lesion-level and prospective evaluations—are warranted to define how DL can be integrated into broader quality-control strategies in teleradiology practice.

## Figures and Tables

**Table 1:** MRI Acquisition Parameters for Dual-Sequence Brain Metastasis Protocol.

Parameter	Skyra 1/2 (Siemens)	Vida (Siemens)	Premier (GE)
**White-Blood (MPRAGE)**			
TR (ms)	1960	1960	2708
TE (ms)	2.55 (2.53 ^†^)	2.55	3.4
TI (ms)	1060	1060	1100
Flip angle (°)	9	9	9
FOV (mm^2^)	240 × 240	240 × 240	220 × 220
Voxel size (mm)	0.75 isotropic	0.75 isotropic	0.8 isotropic
Matrix	320 × 320	320 × 320	278 × 278
Slice thickness (mm)	0.75	0.75	0.8
Acquisition time (min:s)	4:56	4:56	4:46
**Black-Blood (SPACE/CUBE)**			
TR (ms)	530	700	599
TE (ms)	9.3	8.9	22
Flip angle (°)	Variable	Variable	90
FOV (mm^2^)	240 × 240	240 × 240	220 × 220
Voxel size (mm)	0.75 isotropic	0.75 isotropic	0.8 isotropic
Matrix	320 × 320	320 × 320	268 × 268
Slice thickness (mm)	0.75	0.75	0.8
Acquisition time (min:s)	5:41	3:40 *	6:14

**Note.**—* Deep learning-based image reconstruction was applied only to the black-blood SPACE sequence on the VIDA scanner, allowing for reduced scan time without compromising image quality. ^†^ Skyra Room 2 value.

**Table 2:** Patient clinical characteristics and brain metastases information.

Variables	Pre-DL Group (n = 286)	DL-Assisted Group (n = 314)	*p* Value	Effect Size
Age (years)	60.5 ± 15.0	67.2 ± 10.0	<0.001	0.526 ^†^
Sex			0.275	0.048 ^‡^
Female	106 (37.1%)	102 (32.5%)		
Male	180 (62.9%)	212 (67.5%)		
Primary cancer types			NA ^§^	NA ^§^
Lung	239 (83.6%)	260 (82.8%)		
Breast	13 (4.5%)	22 (7%)		
Colorectal	10 (3.5%)	21 (6.7%)		
Renal	5 (1.8%)	11 (3.5%)		
Melanoma	5 (1.8%)	0 (0%)		
Miscellaneous	14 (4.9%)	0 (0%)		
Presence of brain metastases	94 (32.9%)	119 (37.9%)	0.230	0.052 ^‡^
Brain metastases according to primary cancer types			NA ^§^	NA ^§^
Lung	86 (91.5%)	98 (82.4)		
Breast	1 (1.1%)	7 (5.9%)		
Colorectal	2 (2.1%)	8 (6.7%)		
Renal	NA	6 (5%)		
Melanoma	3 (3.2%)	0 (0%)		
Miscellaneous	2 (2.1%)	0 (0%)		
Multiplicity of brain metastases (n = 94)			0.484	0.059 ^‡^
Single	67 (71.3%)	91 (76.5%)		
Multiple	27 (28.7%)	28 (23.5%)		
Size of the largest lesion (n = 94)			0.920	0.007 ^‡^
≤5 mm	70 (74.5%)	88 (73.9%)		
>5 mm	24 (25.5%)	31 (26.1%)		

**Notes:** Data are expressed as the mean ± standard deviation or numbers with percentages in parentheses. *p* value indicates statistical significance between the two groups. ^†^ Effect size calculated using Cohen’s d for continuous variable. ^‡^ Effect size calculated using Cramér’s V for categorical variables. ^§^ NA (not applicable) indicates cancer types not present in the respective study period. DL, deep learning.

**Table 3:** Diagnostic Performance of Teleradiologists: Interpretations Before and After DL Assistance for Brain Metastasis Detection Based on the Reference Standard.

	Sensitivity (%)	Specificity (%)	PPV (%)	NPV (%)	Accuracy (%)
Pre-DL group	77.7 (67.9, 85.6)	82.3 (76.1, 87.4)	68.2 (60.8, 74.8)	88.3 (83.7, 91.7)	80.8 (75.7, 85.2)
Post-DL group	90.8 (84.1, 95.3)	90.8 (85.8, 94.4)	85.7 (79.4, 90.3)	94.2 (90.2, 96.6)	90.8 (87.1, 93.7)
*p*-value ^†^	<0.001	0.002	<0.001	0.011	<0.001

**Notes:** Data in parentheses are 95% confidence intervals. Note. ^†^ *p*-values were calculated using two-tailed Z-tests for independent proportions to compare diagnostic performance between the pre-DL and post-DL groups. DL, deep learning; PPV, positive predictive value; NPV, negative predictive value.

**Table 4:** Individual Survey Responses on DL-assisted Teleradiology Interpretation.

Survey Item	R1	R2	R3	R4	R5	R6	R7	R8	Mean Score
DL assistance was helpful in detecting lesions.	4	5	5	5	5	5	5	5	4.88
DL assistance contributed to reducing interpretation time.	1	5	5	4	4	5	5	4	4.13
There was a high concordance between the DL results and the actual lesion findings.	4	5	4	5	4	5	4	5	4.50
DL tool increased my confidence during interpretation.	4	5	4	4	4	4	5	5	4.38
The visual interface of the DL tool was intuitive and easy to use.	5	5	5	5	5	5	5	5	5.00
Without the DL tool, it would be difficult to maintain the same level of diagnostic accuracy.	2	4	4	2	5	4	4	5	3.75
I would continue to use the DL assistance if it were consistently available in the future.	4	5	5	5	5	5	5	5	4.88

**Notes:** Of the 10 participating radiologists, 8 (80%) completed the post-interpretation survey. Each survey item was rated on a 5-point Likert scale (1 = strongly disagree, 5 = strongly agree) with scores of 2–4 interpreted as indicating varying degrees of agreement or neutrality by eight teleradiologists (R). Mean scores are provided for each item. Items covered lesion detection, workflow impact, confidence, usability, and anticipated future use of DL assistance. DL, deep learning.

## Data Availability

The DL algorithm used in this study is a commercially developed model currently under FDA review, and its trained weights and source code cannot be publicly released due to proprietary restrictions and regulatory review. However, the model configuration and training framework are reproducible based on the cited publications and open-source components. Also, de-identified imaging data (MPRAGE and BB SPACE/CUBE sequences) and summary-level reader performance metrics used in this study are available from the corresponding author upon reasonable request, subject to institutional approval.
